# IL-34 Upregulated Th17 Production through Increased IL-6 Expression by Rheumatoid Fibroblast-Like Synoviocytes

**DOI:** 10.1155/2017/1567120

**Published:** 2017-06-04

**Authors:** Bing Wang, Zijian Ma, Miaomiao Wang, Xiaotong Sun, Yawei Tang, Ming Li, Yan Zhang, Fang Li, Xia Li

**Affiliations:** ^1^Department of Immunology, College of Basic Medical Science, Dalian Medical University, Liaoning, China; ^2^Department of Rheumatology and Immunology, The Third Affiliated Hospital of Hebei Medical University, Hebei, China; ^3^Department of Microecology, College of Basic Medical Science, Dalian Medical University, Liaoning, China; ^4^Department of Rheumatology and Immunology, The Second Affiliated Hospital of Dalian Medical University, Liaoning, China

## Abstract

Rheumatoid arthritis (RA) is a chronic autoimmune disease which is characterized by synovial inflammation and cartilage damage for which causes articular dysfunction. Activation of fibroblast-like synoviocytes (FLS) is a critical step that promotes disease progression. In this study, we aimed to explore the effect of interleukin-34 (IL-34) on RA FLS as a proinflammatory factor and IL-34-stimulated FLS on the production of Th17. We found that serum IL-34 levels were increased compared to those of the healthy controls and had positive correlations with C-reactive protein (CRP), erythrocyte sedimentation rate (ESR), rheumatoid factor (RF), and anticyclic citrullinated peptide (CCP) antibody accordingly. CSF-1R was also highly expressed on RA FLS. The interaction of IL-34 and CSF-1R promoted a dramatic production of IL-6 by FLS through JNK/P38/NF-*κ*B signaling pathway. Further, the IL-34-stimulated IL-6 secretion by RA FLS was found to upregulate the number of Th17. The treatment of IL-6R antagonist could attenuate the production of Th17 mediated by IL-34-stimulated RA FLS. Our results suggest that the increased IL-34 levels were closely related to the disease activity of RA. Additionally, the overexpression of IL-6 in the IL-34-stimulated FLS promoted the generation of Th17. Therefore, IL-34 was supposed to be involved in the pathogenesis of RA. The inhibition of IL-34 might provide a novel target for therapies of RA.

## 1. Introduction

Rheumatoid arthritis (RA) is a progressive systemic disease involving synovial inflammation and articular destruction [1]. In this disease, RA synovial tissue becomes hyperplastic and forms pannus to invade the cartilage and bone, which eventually leads to restricted movement and disability [2]. Fibroblast-like synoviocytes (FLS) play key roles by local production of cytokines and proteolytic enzymes that degrade the extracellular matrix and cartilage only when they are in an activated condition. Accumulating evidence has indicated that many mediators are involved in the RA-FLS activation, such as TNF-*α*, IL-1, IL-6, and so on [3]. These cytokines develop a complex network of autocrine and paracrine regulation mode, which causes not only proliferation and migration but also increased resistance to apoptosis of FLS. And among the cytokines network, there must be new cytokines whose actions are still uncertain. Therefore, understanding the effects and mechanisms of new cytokines underlying in FLS remains a very urgent need.

IL-34 is a newly discovered cytokine, which is consisted of 222 amino acids and shares the same receptor with macrophage colony-stimulating factor (M-CSF) [4, 5], also called colony-stimulating factor-1 receptor (CSF-1R or CD115). In humans, IL-34 mRNA is widely expressed in several of tissues, including heart, brain, lung, liver, kidney, spleen, thymus, testis, ovary, small intestine, prostate, and colon. IL-34 protein was also detected in keratinocytes, epidermis, and neurons [6, 7]. CSF-1R, a member of the platelet-derived growth factor receptor subfamily, is a transmembrane homodimeric type III receptor tyrosine kinase encoded by the c-Fms proto-oncogene [8, 9]. CSF-1R expression is restricted primarily to cells of the mononuclear phagocytic lineage, including macrophage precursors in bone marrow, monocytes, osteoclasts, and tissue macrophages such as liver Kupffer cells and microglia in the brain [10]. It can be also detected on the surface of some tissue fibroblasts [11]. IL-34 binding with CSF-1R is critical for better survival and differentiation of monocytes and macrophages, as well as Langerhans cells (LC) [12]. High expression of IL-34 has been found to correlate with chronic inflammation and some autoimmune diseases such as Sjogren's syndrome (SS) and mucosa of inflammatory bowel disease (IBD) [13, 14]. IL-34 was also upregulated in RA synovium [15, 16], and there was a positive correlation between synovial IL-34 expression and synovitis severity [17]. Cultured RA-derived FLS were showed to produce IL-34 in response to TNF-*α* [18]. One year treatment with disease-modifying antirheumatic drugs (DMARDs) decreased the expression of IL-34 in RA [19]. These studies imply an important role of IL-34 in RA pathogenesis and development. However, it is still not fully clear whether IL-34 can modulate FLS and alter its secretion of proinflammatory cytokines.

In this study, we have observed an increased serum level of IL-34 in RA patients, and it was positively correlated with disease activities. The interaction of IL-34 and CFR-1R significantly enhanced IL-6 production of FLS possibly through JNK/P38/NF-*κ*B signaling pathway. Furthermore, IL-34-stimulated FLS in RA patients upregulated Th17 frequency by increased IL-6 production, and the treatment of IL-6R antagonist could attenuate this effect. Our findings will provide an important implication for better understanding the role of IL-34 in RA inflammatory responses.

## 2. Materials and Methods

### 2.1. Study Population

168 RA patients were recruited (male = 40, female = 128, the average age = 50.29 ± 0.93) from the Department of Rheumatology of the Second Affiliated Hospital of Dalian Medical University, Dalian, China. All patients fulfilled the American College of Rheumatology criteria (ACR 1987). For the control group, 85 healthy volunteers (male = 28, female = 57, the average age = 58.84 ± 1.20) were recruited from the healthy physical center, who were matched by age and gender with the RA subjects (*P* > 0.05). Patients with other systemic diseases and using biological agents and high dose prednisolone were excluded from the study. Experiments were approved by the ethics committee of the Second Affiliated Hospital of Dalian Medical University.

### 2.2. Isolation and Culture of FLS

Synoviocytes were isolated from synovial tissue specimens that were obtained from patients with RA undergoing total joint replacement surgery. Using enzymatic digestion method, the tissue samples were minced into 1-2 mm^3^ pieces and treated with 2.5 mg/ml type I collagenase (Gibco, USA) in Dulbecco's modified Eagle's medium (DMEM) for 2–4 h at 37°C with 5% CO_2_. Dissociated cells were centrifuged for 5 min at 300*g*, and cell pellet was resuspended in DMEM +10% fetal calf serum (FCS) (Gibco, USA), 2 mM L-glutamine, and 100 IU/ml penicillin and streptomycin (Solarbio Life Sciences, China) and seeded in 75 cm^2^ flasks and incubated overnight. Synoviocytes from passages 4–6 were used in each experiment. The cells were morphologically homogeneous and exhibited the appearance of synovial fibroblasts. The purity of cells was tested by flow cytometry using anti-CD14-PE (eBioscience, USA), anti-CD68-APC (Miltenyi Biotec, Germany), and anti-vimentin-ALEXA 488 (BD, USA), and 99% of isolated cells was FLS (CD14^−^ CD68^−^ vimentin^+^).

### 2.3. The Activation of RA FLS by IL-34

5 × 10^5^/ml FLS (*n* = 5) in 10 cm dish were incubated with FBS-free DMEM for 24 hrs, then stimulated with or without IL-34 (50 ng/ml, R&D Systems, USA) for another 24 h, which were prepared for the detection of IL-6 mRNA expression. FLS (1 × 10^5^/ml) were starved in 6-well plate for 24 h, then stimulated with or without IL-34 (50 ng/ml) for 12, 24, 48, and 72 h (*n* = 6) or pretreated with anti-CSF-1R antibody (25 ng/ml, R&D Systems, USA) for 30 min (*n* = 8). The supernatants of cell culture were collected to measure the levels of IL-6.

### 2.4. The Expression of CSF-1R on RA FLS

FLS (2 × 10^5^/ml) (*n* = 6) were cultured in 25 cm^2^ flask and single cell suspensions were collected. Cells were incubated with anti-human FcR block reagent (10 *μ*l/tube) (Miltenyi Biotec, Germany) in PBS containing 1% BSA for 15 min at 4°C and then stained with APC-labeled anti-CSF-1R (5 *μ*g/ml) or mouse IgG2a, *κ* isotype antibody (5 *μ*g/ml) (eBioscience, USA) for 30 min at room temperature. Cells were then washed with PBS twice and resuspended in 500 *μ*l PBS. The expression of CSF-1R on FLS was detected using a flow cytometer (Accuri C6, BD Bioscience, USA) and analyzed by CFlow plus software.

### 2.5. Inhibitors of Signaling Molecules Used in the Experiment

FLS (1 × 10^5^/ml) (*n* = 5) were pretreated with signaling inhibitors (SP600125 (10 *μ*M, inhibitor of JNK), SB203580 (25 *μ*M, inhibitor of P38 MAPK), IKK-16 (10 *μ*M, inhibitor of NF-*κ*B), and FR180204 (10 *μ*M, inhibitors of Erk1/2) (Selleck Chemicals, USA)) for 1 h and stimulated with IL-34 (50 ng/ml) for 24 h or 72 h at 37°C. The mRNA expression level of IL-6 was measured by RT-PCR, and IL-6 production was measured by ELISA.

### 2.6. Western Blotting Analysis

FLS (1 × 10^5^/ml) (*n* = 3 ~ 5) were pretreated with or without signaling inhibitors for 1 h and then stimulated by IL-34 (50 ng/ml) for 30 min, the total protein was extracted from FLS on ice by using Whole Cell Lysis Assay (KeyGEN BioTECH, China) according to manufacturer's protocol, then the protein was gotten after centrifugation at 14,000*g* for 10 min. Western blotting was performed using electrophoresis apparatus (Bio-Rad Co., USA). Briefly, 20 *μ*g of protein in each lane was separated by SDS-PAGE and transferred to polyvinylidene fluoride (PVDF) membranes (Millipore Co., USA). After blocking with 5% skim milk/TBST, the membranes were incubated with primary antibodies overnight at 4°C. The following primary antibodies were used: anti-phospho-JNK antibody (ab124956, Abcam, USA; 1 : 1000), anti-JNK antibody (ab76125, Abcam; 1 : 1000), anti-phospho-NF-*κ*B p65 antibody (ab76302, Abcam; 1 : 2000), anti-NF-*κ*B p65 antibody (ab32536, Abcam; 1 : 2000), anti-phospho-Erk1/2 (ab32538, Abcam; 1 : 500), anti-Erk1/2 antibody (ab184699, Abcam; 1 : 5000), anti-phospho-P38 antibody (ab178867, Abcam, USA; 1 : 1000), and anti-P38 antibody (ab170099, Abcam; 1 : 1000). They were then incubated with the appropriate secondary horseradish peroxidase-conjugated goat anti-rabbit (Abbkine, 1 : 1000) for 2 h. Gel imaging apparatus (Bio-RAD Molecular Imager, ChemiDoc™ XRS+, USA) and analysis software (ImageJ2x, Rawak Software Inc., Germany).

### 2.7. The Coculture of RA FLS and CD4^+^ T Cells of Healthy Controls

RA FLS (2 × 10^4^/ml) were cultured in a 24-well plate overnight. CD4^+^ T cells (2 × 10^5^) were isolated from whole blood of healthy individuals (*n* = 6) using immunomagnetic beads (Miltenyi Biotec, Germany) according to the manufacturer's instructions and then CD4^+^ T cells stimulated with anti-CD3 (3 *μ*g/ml)/CD28 (2 *μ*g/ml) antibody (eBioscience, USA) and brefeldin A (10 ng/ml)/monension (100 ng/ml) (BioLegend, USA) were cocultured with FLS (*n* = 6) for 72 h in the presence or absence of IL-34 (50 ng/ml) in RPMI-1640 medium (Gibco, USA) containing 10% FCS and 1% penicillin-streptomycin solution. Expression frequency of Th17 was measured by a flow cytometer. The IL-6 protein synthesis in the coculture supernatants was detected by ELISA.

To evaluate the effect of IL-6 secreted by FLS on Th17 proportion of CD4^+^ T cell, IL-34-stimulated FLS (*n* = 6) were cocultured by CD4^+^ T cells (*n* = 6) with treatment of anti-CD3 (3 *μ*g/ml)/CD28 (2 *μ*g/ml) antibody and brefeldin A (10 ng/ml)/monension (100 ng/ml) in the presence or absence of IL-6R antagonist (1 *μ*g/ml) (R&D Systems, USA) for 72 h, and single cell suspensions were stained with surface FITC-labeled anti-CD4 (5 *μ*g/ml) (eBioscience, USA) for 30 min and then intracellular stained with APC-labeled anti-IL-17A (5 *μ*g/ml) (eBioscience, USA) for 1 h. Cell staining was completed according to the manufacturer's protocol, and the samples were detected and analyzed using a flow cytometer.

### 2.8. Reverse Transcription PCR (RT-PCR)

Total RNA was extracted from cultured FLS (*n* = 5) pellets using RNAisoPlus (Takara Bio, Japan) and then the quantity and purity of RNA was examined by checking A260/A280 and agarose gel electrophoresis. Reverse transcription of 2 *μ*g of total mRNA was performed using a PrimeScript™ 1st Strand cDNA Synthesis Kit (Takara Bio, Japan), and the resulting cDNA was subjected to PCR amplified for 33 cycles. Each cycle included 30 s of denaturation at 95°C, 30 s of annealing at 56°C, and 30 s of extending at 72°C. GAPDH was amplified as an internal control. The primers used in this study were listed as follows: GAPDH (272 bp): ACCACAGTCCATGCCATCAC (forward), CGCCTGCTTCACCACCTTCTT (reverse). IL-6 (234 bp): CCTTCGGTCCAGTTGCCTTCTC (forward), CCAGTGCCTCTTTGCTGCTTTC (reverse). PCR products of each gene were then observed by electrophoresis on 2% agarose gels.

### 2.9. Enzyme-Linked Immunosorbent Assay (ELISA)

IL-6 and IL-34 levels were measured by ELISA kit (BioLegend, USA) according to the manufacturer's instructions. Sensitivity: the minimum detectable concentration of IL-6 for this set is 4 pg/ml, and the minimum detectable concentration of IL-34 is 33.4 pg/ml.

### 2.10. Serum Profiling on Protein Chip Assay

FLS from RA synovium (*n* = 5) were treated with or without IL-34 (50 ng/ml) for 72 h. The levels of cytokines in supernatants were detected by Protein chip AAH-CYT-G1000 Kit (RayBiotech, Norcross, GA) in accordance with the instructions. InnoScan 300 Microarray Scanner (31390 Carbonne, France) was used for the signal scanning, the median foreground, and the background intensities for each spot in the protein microarrays were obtained and analyzed with AAH-CYT-G6 and AAH-CYT-G7 software. Sensitivity: the minimum detectable concentration is 1 pg/ml.

### 2.11. Statistical Analysis

All data are expressed as the mean ± standard error of the mean (SEM). Statistical comparison between the two groups was analyzed by paired *t*-test comparisons or Wilcoxon signed rank test; potential correlations between variables were examined using Spearman's rank correlation through GraphPad Prism 5 (San Diego, CA, USA). Statistical significance was obtained with *P* values <0.05.

## 3. Results

### 3.1. Elevated Serum IL-34 Levels Were Positively Correlated with Disease Activities in RA Patients

**Figure 1:**
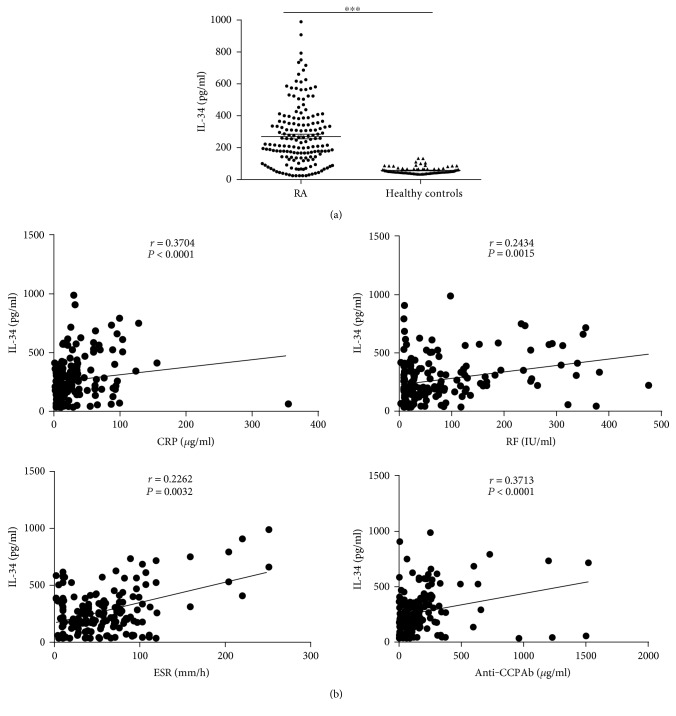
Serum IL-34 levels in RA patients were elevated and correlated with CRP, ESR, RF, and anti-CCP antibody levels. (a) Serum concentrations of IL-34 were measured by ELISA in RA patients (*n* = 168) and healthy controls (*n* = 85). Data were expressed as the mean ± SEM. ^∗∗∗^*P* < 0.0001. (b) The IL-34 concentrations of RA patients were correlated significantly with CRP (*r* = 0.3704, *P* < 0.0001), ESR (*r* = 0.2262, *P* = 0.0032), RF (*r* = 0.2434, *P* = 0.0015), and anti-CCP antibody (*r* = 0.3713, *P* < 0.0001) (*n* = 168). Correlations between variables were examined using Spearman's rank correlation.

We quantified the serum IL-34 levels comparison between 168 RA patients and 85 healthy people by ELISA. These patients' characteristics and drug use situation were shown in Table 1. Amount of IL-34 was significantly higher in RA patients (269.72 ± 14.71 pg/ml) compared to that in healthy controls (56.74 ± 2.30 pg/ml) (Figure 1(a)). In addition, elevated IL-34 was found to positively correlate with CRP, ESR, RF, and anti-CCP antibody (Figure 1(b)), but not with other laboratory indexes including amount of IgA, IgG, IgM, IgE, C3, and C4 (Table 2). These results demonstrate that an overexpression of serum IL-34 in RA patients may be associated with the disease process.

### 3.2. The Interaction of IL-34 and IL-34 Receptor Promoted the Production of IL-6 by FLS

**Figure 2:**
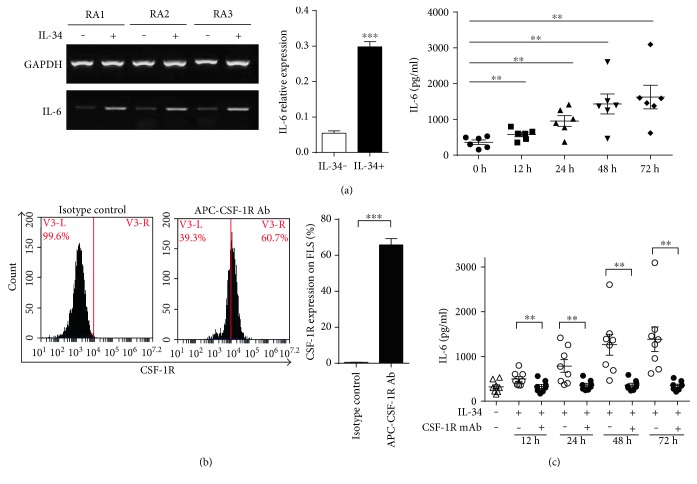
The interaction of IL-34 and CSF-1R promoted the production of IL-6 by RA FLS. (a) RA FLS (*n* = 5) were stimulated with IL-34 (50 ng/ml) for 24 h. IL-6 mRNA expression was measured by RT-PCR (left); data represents the mean ± SEM, ^∗∗∗^*P* < 0.001 compared to the control group (middle). RA FLS (*n* = 6) were incubated with 50 ng/ml IL-34 for 0, 12, 24, 48 and 72 h. IL-6 levels in the supernatants were measured by ELISA. Data represents the mean ± SEM, ^∗∗^*P* < 0.01 (right). (b) Cultured RA FLS (*n* = 6) were stained with APC-labeled anti-CSF-1R (5 *μ*g/ml) or isotype control antibody (5 *μ*g/ml). CSF-1R expression was detected by flow cytometric analysis. Data represents the mean ± SEM. ^∗∗∗^*P* < 0.0001 compared with the isotype control antibody group. (c) RA FLS (*n* = 8) were stimulated with IL-34 (50 ng/ml) for 0, 12, 24, 48, and 72 h after 30 min preincubation with 25 ng/ml anti-CSF-1R mAb or not. Data represents the mean ± SEM. ^∗∗^*P* < 0.01 compared with the group incubated with anti-CSF-1R mAb.

Evidences showed that RA FLS could keep an excited state for a long time once they were stimulated by proinflammatory mediators and continued to produce a broad range of cytokines including IL-6. In this experiment, we have tested whether IL-34 affects IL-6 synthesis by FLS in RA patients. FLS from six RA patients were stimulated with 50 ng/ml of IL-34 for 24 h, and we found that IL-6 mRNA expression was significantly higher in IL-34-stimulated FLS by RT-PCR analysis. In addition, the IL-34-stimulated FLS produced remarkably higher IL-6 compared to unstimulated FLS (Figure 2(a)). Our results suggest that IL-34 had an inflammatory influence on RA FLS.

In order to investigate whether the enhanced IL-6 is produced by the binding of IL-34 with CSF-1R, we analyzed the expression of CSF-1R on FLS by using flow cytometry. The result showed that CSF-1R was highly expressed on FLS in RA patients (Figure 2(b)). In addition, the anti-CSF-1R mAb had an antagonistic effect on IL-6 production when FLS were preincubated with anti-CSF-1R mAb (25 ng/ml) in the presence of IL-34 (50 ng/ml) for 0, 12, 24, 48, and 72 h (Figure 2(c)), which suggests that the interaction of IL-34 with CSF-1R promoted IL-6 production by RA FLS.

### 3.3. IL-34 Dramatically Promoted IL-6 Production of FLS via JNK/P38/NF-*κ*B Signaling Pathways

**Figure 3:**
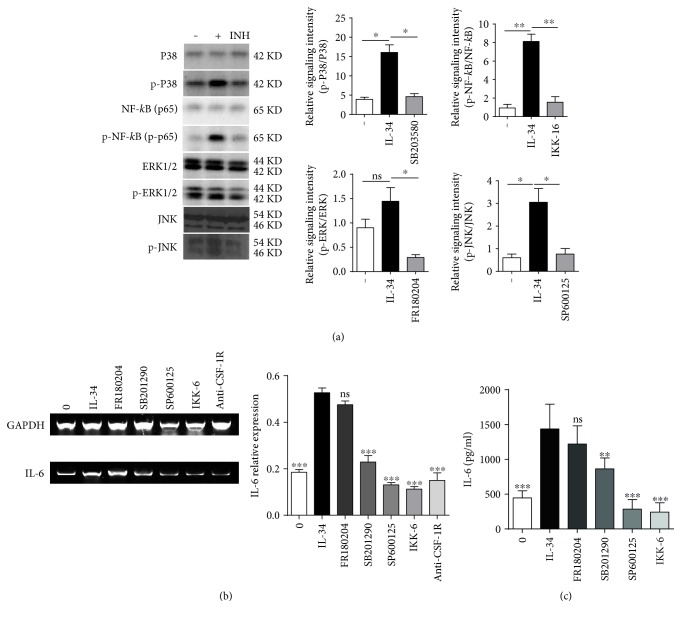
IL-34 dramatically promoted IL-6 production of FLS through JNK/P38/NF-*κ*B signaling pathway. (a) RA FLS (*n* = 3 ~ 5) were pretreated with or without inhibitors of signaling molecules for 1 h and then stimulated by IL-34 (50 ng/ml) for 30 min. Protein was acquired in whole cell lysis buffer (20 *μ*g/lane); meanwhile, phosphorylations of P38, NF-*κ*B, ERK1/2, and JNK were analyzed by Western blotting using anti-phospho-specific antibody. Total P38, NF-*κ*B, ERK1/2, and JNK (20 *μ*g/lane) were determined by Western blotting using corresponding antibodies, respectively. The expression ratios of p-P38 to P38, p-NF-*κ*B to NF-*κ*B, p-ERK1/2 to ERK1/2, and p-JNK to JNK were represented in a bar graph. Data represents the mean ± SEM. ^∗^*P* < 0.05, ^∗∗^*P* = 0.0074, ns: no significant compared with the untreated cells. (b) FLS isolated from RA patients (*n* = 5) were pretreated with inhibitors of signaling molecules for 1 h or pretreated with anti-CSF-1R mAb (25 ng/ml) for 30 min, respectively, and then incubated with IL-34 (50 ng/ml) for 24 h. The expression of IL-6 mRNA was detected by RT-PCR. Data represents the mean ± SEM. ^∗∗∗^*P* < 0.0005, ns: no significant compared to the group incubated with IL-34 only. (c) FLS isolated from RA patients (*n* = 5) were pretreated with inhibitors of signaling molecules for 1 h and then incubated with IL-34 (50 ng/ml) for 72 h. IL-6 levels in the supernatants were measured by ELISA. Data represents the mean ± SEM. Statistical analysis was using the paired *t*-test. ^∗∗∗^*P* < 0.0001, ^∗∗^*P* = 0.0017, ns: no significant compared with the IL-34 group.

Previous studies have demonstrated that some inflammatory diseases are associated with dysregulation of the IL-34/CSF-1R axis in humans. IL-34 stimulation might promote phosphorylation of several tyrosine residues in the kinase domain of CSF-1R. In the current study, we would like to confirm whether IL-34 induced IL-6 production through MAPKs and NF-*κ*B signal pathways that have been known to associate with cell proliferation. Results showed that the phosphorylation of P38, NF-*κ*B, and JNK but not ERK1/2 in the cytoplasm of FLS was significantly increased after FLS was treated with IL-34 (50 ng/ml) for 30 min. Further, when RA FLS were stimulated with four signal molecules' inhibitors including SP600125, SB203580, FR180204, and Ikk-16, which inhibited JNK, P38 MAPK, ERK1/2, and NF-*κ*B activation, respectively, the phosphorylation of P38, NF-*κ*B, ERK1/2, and JNK was suppressed apparently (Figure 3(a)). Accordingly, the expression of IL-6 mRNA and protein in IL-34-stimualted FLS were significantly decreased in the presence of SP600125, SB203580, and IKK-6, but FR180204 impaired the production of IL-6 only to a small extent (Figures 3(b) and 3(c)). Based on these results, it is concluded that IL-34 induced IL-6 production possibly through JNK/P38 MAPK and NF-*κ*B signaling pathways.

### 3.4. IL-34 Upregulated Th17 Production through Increased IL-6 Expression by RA FLS

**Figure 4:**
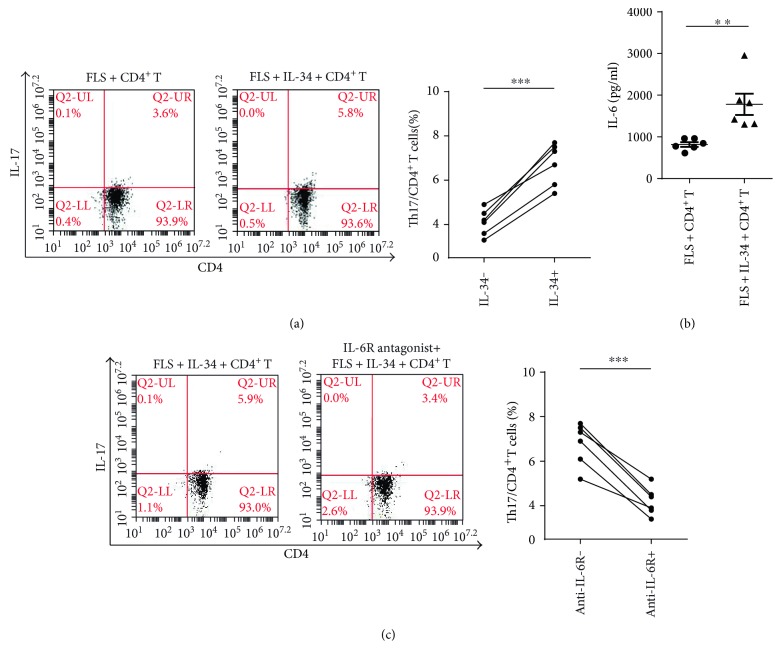
IL-34 upregulated the production of Th17 through increased IL-6 expression by RA FLS. (a) RA FLS and CD4^+^ T cells from healthy controls (*n* = 6) were incubated with anti-CD3 (3 *μ*g/ml)/CD28 (2 *μ*g/ml) antibody, brefeldin A (10 ng/ml)/monension (100 ng/ml), and IL-34 (0 or 50 ng/ml) for 72 h, CD4^+^ IL-17^+^ T cells were analyzed by flow cytometric analysis. (b) CD4^+^ T cells and RA FLS (*n* = 6) were cocultured with anti-CD3 (3 *μ*g/ml)/CD28 (2 *μ*g/ml) antibody and IL-34 (0 or 50 ng/ml) for 72 h. IL-6 levels in the supernatants of coculture system were measured by ELISA. (c) RA FLS and CD4^+^ T cells (*n* = 6) were preincubated with anti-IL-6R antibody (1 *μ*g/ml) for 1 h and then stimulated with anti-CD3 (3 *μ*g/ml)/CD28 (2 *μ*g/ml) antibody, brefeldin A (10 ng/ml)/monension (100 ng/ml), and IL-34 (50 ng/ml) for 72 h. CD4^+^ IL-17^+^ T cells were analyzed by flow cytometry. Data represents the mean ± SEM. ^∗∗∗^*P* < 0.001, ^∗∗^*P* < 0.01.

As IL-6 is critical in maintaining a balanced Th17 immune response, and an elevated IL-6 was observed in IL-34-stimulated FLS in RA patients; we then tested whether this increased IL-6 by FLS could alter Th17 production. We have detected that T and B lymphocytes did not express CSF-1R (data not shown), so we cultured RA FLS with purified CD4^+^ T cells in a 24-well plate together and stimulated them by IL-34. Results showed that the amounts of Th17 in the IL-34-stimulated coculture system were higher compared to that of the unstimulated system, accompanied by an increased IL-6 (Figures 4(a) and 4(b)). However, the elevated Th17 response was impaired when anti-IL-6R antibody was added into the coculture system (Figure 4(c)). These data indicate that IL-34-stimulated FLS facilitated Th17 generation via increased IL-6 production in vitro.

### 3.5. Chemokines Expression on IL-34-Stimulated FLS in RA Patients

**Figure 5:**
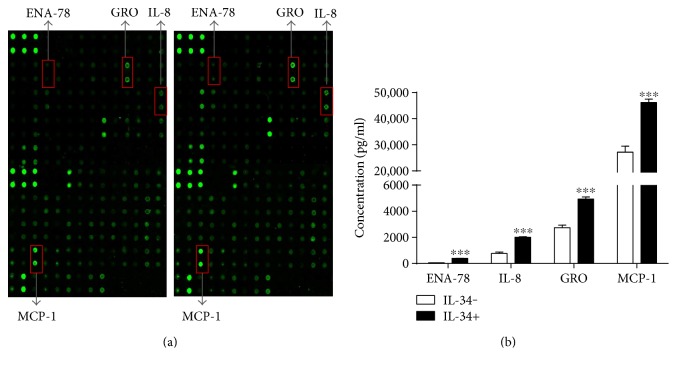
IL-34 increased the expression of inflammatory chemokines in RA FLS. RA FLS (*n* = 5) were stimulated with or without IL-34 (50 ng/ml) for 72 h, and the supernatants of RA FLS were detected with the protein chip AAH-CYT-G1000 kits. (a) Four chemokines (ENA-78, GRO, IL-8, and MCP-1) with obvious enhanced brightness after being incubated with IL-34 (right part) for 72 h were picked out for further analysis. (b) The secretion levels of the four chemokines were analyzed in RA FLS supernatant with or without IL-34 stimulation. Data represents the mean ± SEM. ^∗∗∗^*P* < 0.001.

We also tested whether IL-34 stimulation affected the expression of some chemokines. By using protein chip AAH-CYT-G1000 kits, we found that IL-34-stimulated FLS exhibited significantly increased epithelial neutrophil-activating peptide-78 (ENA-78), interleukin-8 (IL-8), growth-related oncogene (GRO), and macrophage chemoattractant protein-1 (MCP-1) compared to the unstimulated control (Figures 5(a) and 5(b)).

## 4. Discussion

Some researchers have revealed that IL-34 has a role in immunological regulation, proinflammation, and nerve protection. It has been confirmed that IL-34 is secreted by neurons, keratinocytes, and osteoblasts; also, FLS in RA patients produce IL-34 following TNF-*α* and IL-1*β* stimulation [18, 19]. The elevated IL-34 in serum has been observed in RA patients, which is associated with radiographic progression, RF, and anti-CCP antibody titers [20, 21]. IL-34 greatly increases the activation and proliferation of osteoclast, suggesting that IL-34 may have a potential role in bone destruction in RA [22]. In the current study, we confirmed that IL-34 was overexpressed in RA patients and had a substantial correlation with disease activities.

Meanwhile, CSF-1R was highly expressed on RA FLS. The IL-34/CSF-1R axis promoted a dramatic production of IL-6 by FLS through JNK/P38/NF-*κ*B signaling pathways. Further, IL-6 secreted by IL-34-stimulated RA FLS was found to upregulate the number of Th17.

IL-6 is a pleiotropic cytokine, which is produced mainly by lymphocytes, macrophages, epithelial cells, tumor cells, and also FLS in humans [23]. Accumulated evidence potentiated that FLS can be activated under inflammation. Here, we showed that IL-34 could promote FLS to produce IL-6 in RA patients. IL-6 can not only contribute to the production of matrix metalloproteinases (MMPs) but also induce the differentiation and activation of osteoclasts [24]. IL-6 can also lead to human Th17 generation [25, 26]. In our study, we found that IL-34-stimulated FLS in RA patients could enhance the numbers of Th17 and addition of IL-6R antagonist reversed Th17 response, which indicated that the combination of IL-6/IL-6R mediated Th17 production in CD4^+^ T cells. Our data suggests that IL-34 could have direct effects on the secretion of IL-6 by FLS as an upstream proinflammatory cytokine. Also, IL-34 was able to upregulate Th17 production through the overexpressed IL-6 on FLS in RA patients.

IL-34 acts on macrophages and Langerhans cells upon binding CSF-1R [12]. CSF-1R is mainly found in myeloid cells. In the present study, we also got the results that CSF-1R was highly expressed on FLS and IL-34 might affect the function of FLS via binding with CSF-1R. CSF-1R signaling pathways are often involved in the survival, proliferation, and differentiation of myeloid cells, and there are a wide variety of downstream proteins participating in the process [10, 27]. JNK/P38 MAPK/ERK1/2 signal pathways take part in cell cycle regulation and cell apoptosis. Furthermore, NF-*κ*B pathway, an important transcription factor, is involved in the regulation of gene transcription related to immune response, inflammation, and cell differentiation [28–31]. In this study, we found that IL-34 induced the phosphorylation of P38, NF-*κ*B, and JNK compared with the controls. However, the phosphorylation of ERK1/2 itself kept a relatively high level in the cytoplasm of RA FLS when they were not treated with IL-34. Additionally, the amounts of IL-6 secreted by IL-34-stimulated RA FLS were decreased in the presence of SB201290, SP600125, andIKK-6, except for FR180204. These data indicated that IL-34/CSF-1R axis favored the generation of IL-6 via JNK/P38 MAPK/NF-*κ*B pathways, but not ERK1/2.

CSF-1R signaling pathway also mediates the chemotaxis of microglia and monocytes [32]. Several chemokines and their receptors were implicated in FLS infiltration, macrophage recruitment, and angiogenesis in RA [33]. Our results by protein chips showed that the levels of ENA-78, IL-8, GRO, and MCP-1 were significantly increased in the FLS culture supernatants after IL-34 stimulation. ENA-78, IL-8, and GRO belong to the C-X-C subgroup. They can combine with CXCR2 then promote neutrophil attachment and exudation, mediate the inflammation and immune mediation, and delay apoptosis [34, 35]. MCP-1 belongs to the CC subgroup, whose receptors are CCR5 and CCR2. In most cases, it can bind with CCR2 specifically. MCP-1 has the function of chemotaxis and activation of monocytes, macrophages, and T lymphocytes [36]. The findings that IL-34 could promote the production of some chemokines associated with inflammation and autoimmune diseases indicated that IL-34 might play multiple roles in the pathology of RA.

In summary, we found that IL-34 could combine with CSF-1R on RA FLS to elevate the expression of IL-6, which subsequently promoted the Th17 production. Therefore, IL-34 might constitute a key cytokine that affect the interactions between inflammatory cells in RA. Targeting of IL-34 might represent a useful therapeutic strategy for RA. However, the mechanism about how IL-34 reacts with FLS and T cells will need further exploration.

## Figures and Tables

**Table 1:** Baseline clinical characteristics and medication of RA patients ($\bar{x}$ ± SEM or *n*%).

	Baseline value
Age, year	50.29 ± 0.93
Male/female	40/128
Disease duration, years	9.62 ± 2.23
DAS28	4.41 ± 1.39
ESR, mm/h	55.69 ± 3.68
CRP, *μ*g/ml	33.84 ± 3.50
RF, IU/ml	79.01 ± 7.39
Anti-CCP Ab, U/ml	173.80 ± 18.35
Current NSAID users	4 (2.38)
Current DMARD users	21 (12.50)
Current steroid users	2 (11.90)
Two-type drug users	67 (39.88)
Three-type drug users	15 (8.93)
No systemic therapy patients	59 (35.11)

ESR: erythrocyte sedimentation rate; CRP: C-reactive protein; DAS: disease activity score; RF: rheumatoid factor; CCP: cyclic citrullinated peptide; NSAIDs: nonsteroidal anti-inflammatory drugs; DMARDs: disease-modifying antirheumatic drugs.

**Table 2:** Correlation analysis for IL-34 and other clinical data in patients with RA.

Variables	*n*	Spearman rank correlation coefficient (*r*)	*P*
IgA	146	0.01771	0.8320
IgG	147	0.03583	0.6666
IgM	145	0.06114	0.4650
IgE	146	0.06142	0.4614
C3	146	0.02767	0.7402
C4	146	−0.07713	0.3548
